# Evaluation of the impact of filter types and parameters upon the accuracy of phase-based optical flow method with a complex steerable pyramid

**DOI:** 10.1371/journal.pone.0308943

**Published:** 2024-09-04

**Authors:** Zhaoxin Peng, Xian Wang, Zhiliang Wang, Wei Liu, Menglian Liu

**Affiliations:** 1 School of Mechanical Engineering, Hunan University of Science and Technology, Xiangtan, China; 2 Engineering Research Centre of Hunan Province for the Mining and Utilization of Wind Turbines Operation Data, Hunan University of Science and Technology, Xiangtan, China; Islamic Azad University, ISLAMIC REPUBLIC OF IRAN

## Abstract

Complex steerable pyramid (CSP) performs well when applied to magnify subtle motions of structures for observing the dynamic characteristics of facilities. However, the impact of the types and parameters of CSP filters upon the performance of phase-based optical flow (PBOF) in measuring motion parameters has not been systematically studied. The purpose of this study is to comprehensively evaluate the impact of different CSP filter types (Octave, HalfOctave, SmoothHalfOctave, and QuarterOctave) and parameters on the performance of PBOF in measuring motion parameters. Firstly, by measuring simulated translational motion, the influence of the CSP’s down-sampling rates on the displacement measurement accuracy of PBOF is analyzed to determine appropriate settings. Subsequently, the effective displacement measurement interval and accuracy of PBOF using the CSP are studied through simulated and experimental translational motion measurements. Further, the vibration parameter’s accuracy is analyzed through simulated periodic vibration measurements. Finally, the characteristics of PBOF using the four kinds of CSP and practical considerations are discussed. Simulation and experimental results demonstrate that when using middle-level filters within the effective level range of HalfOctave, PBOF achieves the best overall displacement measurement performance. Additionally, this method can easily integrate with signal processing techniques in analyzing structural dynamic characteristics under field conditions.

## 1. Introduction

The displacement signals, such as vibration, dynamic deflection, and dynamic deformation, generated during the operational processes of equipment and structures contain rich inherent structural features and system state information. These signals form the foundational data for condition monitoring, safety assessment, life prediction, and structural optimization. Consequently, structural displacement measurement technology has consistently remained a focal point of attention [1].

In recent years, when compared with the contact measurement method [2–4] and the non-contact measurement method [5–7] based on the theory of light wave interference and laser Doppler effect, the rapidly growing visual displacement measurement technology [8–11] has emerged as the most prominent technology in the field of structural displacement measurement due to its significant advantages. These advantages include high efficiency, low equipment costs and site layout costs, and high spatial resolution in measurement.

Visual displacement measurement methods can be divided into two categories: methods based on image grayscale and methods based on image phase [12]. Image grayscale is obtained through the camera sensor’s perception of the object’s illumination intensity, while phase is acquired by convolving the image with a pair of orthogonal complex-valued filters. The methods based on image grayscale have been widely used in structural displacement measurement [13, 14], such as the feature point tracking [15, 16], the digital image correlation (DIC) [17, 18], and the gradient-based optical flow (GBOF) [19, 20]. For example, Lydon et al. [21] and Zhu et al. [22] proposed a combination of feature points and GBOF to measure the dynamic displacement of structures. Wei et al. [23] proposed a 3D-DIC method to measure the full-field dynamic displacement of the cassette structure.

In contrast to the GBOF, the phase-based approach demonstrates enhanced resistance to interference and greater adaptability to changes in illumination and viewing angles [24, 25]. Since Wadhwa et al. [26] proposed the phase-based motion magnification technique using the complex steerable pyramid (CSP) filter decomposition image algorithm [27, 28] to observe subtle motions, the image phase-based displacement measurement method has gained increasing attention. The CSP filter decomposition image algorithm was proposed by Wadhwa et al. when studying video motion magnification. This method decomposes images to obtain the spatial phase information of different frequency domain sub-bands and ensures that there is no aliasing among the frequency domain sub-bands, so that high-quality motion magnified images can be reconstructed. The core principle of video motion magnification is to use the CSP filter decomposition image algorithm to obtain the motion phases of the video, magnify the phases and reconstruct the original video to visualize small displacements. In order to further increase the magnification factor of motion magnified videos and improve image quality, Wadhwa et al., based on Octave CSP, successively proposed HalfOctave, SmoothHalfOctave and QuarterOctave CSP. Among these CSPs, which use narrower filter bandwidths to achieve higher magnification, SmoothHalfOctave and QuarterOctave CSP use smoother filters to improve the quality of motion magnified images. Chen et al. [29] first proposed combining phase-based optical flow (PBOF) with CSP to measure the vibration of a cantilever beam. In the PBOF using CSP filter, the CSP filter decomposition image algorithm is used to obtain the motion phases of images. Based on the phase constancy assumption, PBOF calculates the displacement of the object from the obtained motion phase. This process does not involve the magnification or reconstruction of any motion phase of a video. Therefore, unlike motion magnification of a video, although PBOF also uses the CSP filters decomposition image algorithm to obtain the motion phases of images, it does so to accurately measure the displacement, not just visualize small displacements. Then, various techniques have been developed to utilize the image phase obtained through CSP decomposition for motion estimation, structural health monitoring, and defect detection of structures. Cosco et al. [30] proposed a defect detection method for vibrating plates based on PBOF using CSP. Jana et al. [31] realized the practical estimation of the real-time vibration frequency of the bridge by combining phase-based motion estimation using CSP with the signal blind source separation method.

A deep understanding of the performance characteristics of various visual displacement measurement methods is the basis for successfully using these methods in engineering practice. Diamond et al. [32] evaluated the micro-displacement measurement accuracy of PBOF and analyzed the impact of the measured pixel position and image down-sampling ratio upon the PBOF measurement accuracy. Based on the above work, Collier et al. [33] studied the interval of effective displacement measurement of PBOF and analyzed the impact of image contrast and bit depth upon the range of effective displacement measurement. The above fruitful studies provide a better understanding of the characteristics of the PBOF algorithm to a certain extent and help promote the application of the PBOF algorithm. However, to the best of the author’s knowledge, there is currently no systematic study on the impact of CSP filter types and parameters on the performance of PBOF in measuring motion parameters, and such an absence leads to a lack of reliable basis in engineering practice for selecting CSP filter types and parameters according to specific circumstances.

The purpose of this study is to comprehensively evaluate the impact of different CSP filter types (Octave, HalfOctave, SmoothHalfOctave, and QuarterOctave) and parameters on the performance of PBOF in measuring motion parameters. This evaluation is conducted through simulations of translational motion, periodic vibration, and experimental translational motion. The evaluation metrics of the measurement performance of motion parameters include the effective displacement measurement interval and accuracy, and the accuracy of measuring the vibration frequency. In addition, the characteristics of PBOF using four kinds of CSP and the problems that need attention in practical applications are discussed.

## 2 Theories

### 2.1 Overall framework

**Fig 1:**
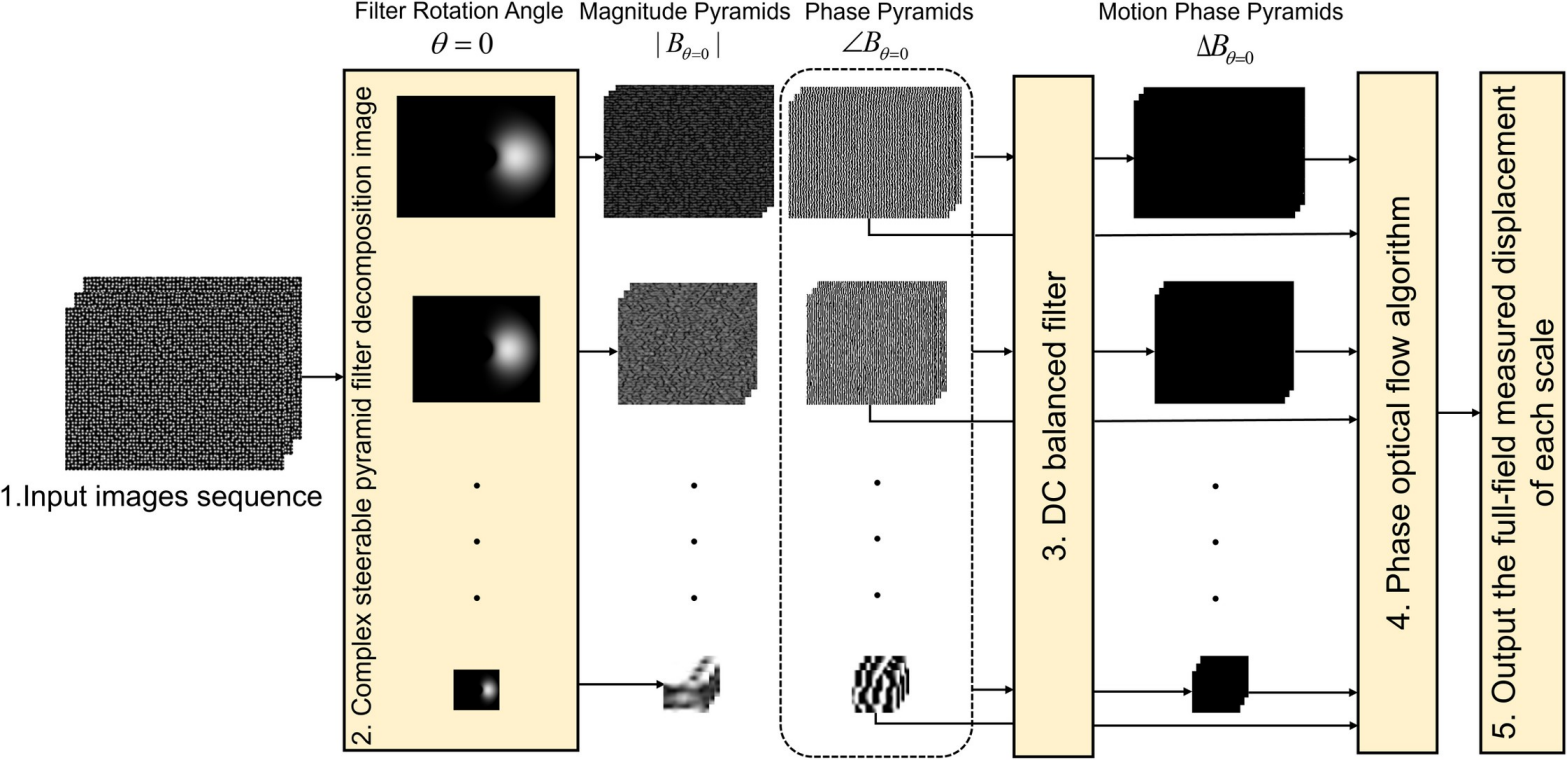
The overall framework of the PBOF using the CSP. The complex filter rotation angle *θ* = 0.

The implementation of the PBOF using CSP in this study is based on reference [29], and the overall framework of this method is shown in Fig 1. First, the spatial domain amplitude and phase information of each frame of the input image sequence in the *x* and *y* directions are extracted by using the complex filter *G*_*θ*_ + *iH*_*θ*_ with rotation angles *θ* being 0 and π/2 respectively in the CSP at different image scales:

$$R\left(x,y,t_{j}\right)=A_{\theta }\left(x,y,t_{j}\right)e^{i\phi_{\theta }\left(x,y,t_{j}\right)}=\left(G_{\theta }+iH_{\theta }\right)*I\left(x,y,t_{j}\right)\;x,y\in R$$ (1)

In the Eq (1) , *I*(*x*,*y*,*t*_*j*_) is the grayscale of the pixel when the coordinates are (*x*,*y*) at time *t*_*j*_; * is convolution computation; *R*(*x*,*y*,*t*_*j*_) is the spatial domain response of the image to the complex filter *G*_*θ*_ + *iH*_*θ*_ in the *θ* direction at time *t*_*j*_, and *A*_*θ*_ (*x*,*y*,*t*_*j*_) and *ϕ*_*θ*_ (*x*,*y*,*t*_*j*_) are the spatial domain amplitude and phase response respectively.

According to the assumption of constant phase, the phase of the same pixel remains unchanged in adjacent images:

$$\phi_{\theta }(x,y,t)=\phi_{\theta }(x+dx,y+dy,t+dt)$$ (2)

The Taylor series expansion of $\phi_{\theta }(x+dx,y+dy,t+dt)$ is carried out at (*x*,*y*,*t*), and the first-order derivative approximation is taken. Eq (2) is transformed into:

$$\frac{\partial \phi_{\theta }}{\partial x}dx+\frac{\partial \phi_{\theta }}{\partial y}dy+\frac{\partial \phi_{\theta }}{\partial t}dt=0$$ (3)

The gradient of *ϕ*_*θ*_ (*x*,*y*,*t*) at the spatial coordinates (*x*,*y*), $\nabla \phi_{\theta }(x,y,t)={\left(\frac{\partial \phi_{\theta }}{\partial x},\frac{\partial \phi_{\theta }}{\partial y}\right)}^{\text{T}}:$

$$\nabla \phi_{\theta }(x,y,t)=\frac{Im\left[R^{*}(x,y,t)\nabla R(x,y,t)\right]}{A_{\theta }^{2}(x,y,t)}$$ (4)

where R^*^(*x*,*y*,*t*) is the conjugate of *R*(*x*,*y*,*t*); $\nabla R(x,y,t)={\left(\frac{\partial R}{\partial x},\frac{\partial R}{\partial y}\right)}^{\text{T}}$ is the gradient of *R*(*x*,*y*,*t*) at the spatial coordinates (*x*,*y*) and the operator *Im*[•] is the imaginary part of the complex number. The partial derivative $\frac{\partial \phi_{\theta }}{\partial t}$ of *ϕ*_*θ*_ to the time variable *t* can be obtained by using forward difference:

$$\frac{\partial \phi_{\theta }}{\partial t}=\phi_{\theta }(x,y,t)-\phi_{\theta }\left(x,y,t_{j}\right)$$ (5)

Let the instantaneous velocity vector of the pixel at the spatial coordinates (*x*,*y*) be ***v*** = (*v*_*x*_,*v*_*y*_)^Τ^ and then Eq (3) can be transformed into:

$$\frac{\partial \phi_{\theta }}{\partial x}v_{x}+\frac{\partial \phi_{\theta }}{\partial y}v_{y}+\frac{\partial \phi_{\theta }}{\partial t}=0$$ (6)

When *θ* = 0, the partial derivative of *ϕ*_0_ (*x*,*y*,*t*) to the coordinate variable *y* is $\frac{\partial \phi_{0}}{\partial y}\approx 0$. When *θ* = π/2, the partial derivative of *ϕ*_π/2_(*x*,*y*,*t*) to the coordinate viriable *x* is $\frac{\partial \phi_{\pi /2}}{\partial x}\approx 0$. The following formula can be obtained by substituting the above conditions into Eq (6) respectively:

$$v={\left(v_{x},v_{y}\right)}^{T}={\left(-{\left(\frac{\partial \phi_{0}}{\partial x}\right)}^{-1}\frac{\partial \phi_{\pi /2}}{\partial t},-{\left(\frac{\partial \phi_{\pi /2}}{\partial y}\right)}^{-1}\frac{\partial \phi_{\pi /2}}{\partial t}\right)}^{T}$$ (7)

By multiplying the velocity vector **v** with the time interval between the corresponding two frames Δ*t*, the displacement vector **d** of the pixel at (*x*,*y*) can be obtained:

$$d=v\Delta t={\left(d_{x},d_{y}\right)}^{T}$$ (8)

where *d*_*x*_ and *d*_*y*_ are the displacement of the spatial coordinates (*x*,*y*) along the *x* and *y* directions.

According to the literature [34, 35], when using the PBOF to obtain the full-field measurement displacement of all frames, the pixels *I*(*x*,*y*,*t*) in the image may randomly exhibit a non-linear phase response, leading to significant measurement errors at that point. To address this issue, literature [34, 35] proposes using non-linear phase response confidence to filter pixels for motion estimation. This method is implemented when an adequate number of frames are available. Therefore, the above method cannot select pixel positions that can be accurately measured for PBOF in a single frame measurement. In order to eliminate the potential interference of pixel position selection on the measurement accuracy of PBOF, this study adopts the method from reference [33] to obtain the final measurement result of PBOF in a single frame image. When using PBOF with a CSP filter, for each frame image, *n* measurement values **d** will be obtained, where *n* represents the total number of pixels in the single frame image. According to the three-sigma rule, values within the ***μ* ± 3σ** interval are selected as effective measurement values from the *n* measurement values, and the average value ***m*** of the effective measurement values is taken as the final measurement result of PBOF for the single-frame image.

### 2.2 Complex steerable pyramid

**Fig 2:**
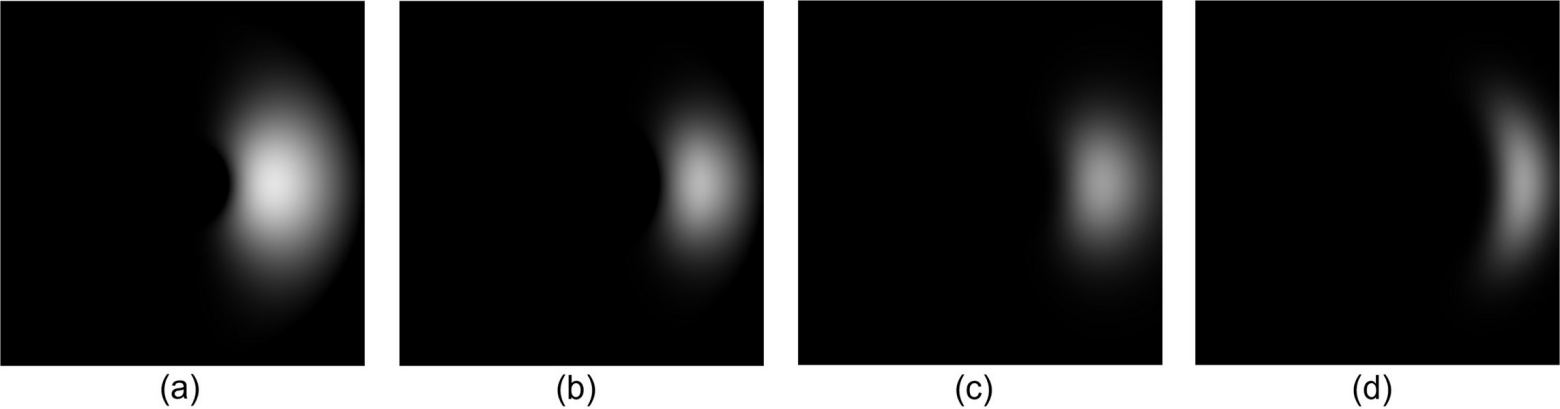
The oriented band-pass filters of the four types of CSP at level 1 and rotation angle 0. (a) Octave. (b) HalfOctave. (c) SmoothHalfOctave. (d) QuarterOctave.

In the overall framework of PBOF using CSP, the function of the CSP is to decompose the image so that the multi-scale spatial domain phase information in the directions of θ = 0 and θ = π/2 can be obtained (corresponding to step 2 of Fig 1). There are four types of CSP including Octave, HalfOctave, SmoothHalfOctave, and QuarterOctave [26]. The processes of decomposing images of these four types of CSP are similar, while the difference lies in the high-pass filter, low-pass filter, and oriented band-pass filter used in the image decomposition process. In the CSP filter decomposition image algorithm, the oriented band-pass filters play a core role. The oriented band-pass filters of the four different types of CSP at level 1 and rotation angle 0 are shown in Fig 2.

#### 2.2.1 Octave CSP

**Fig 3:**
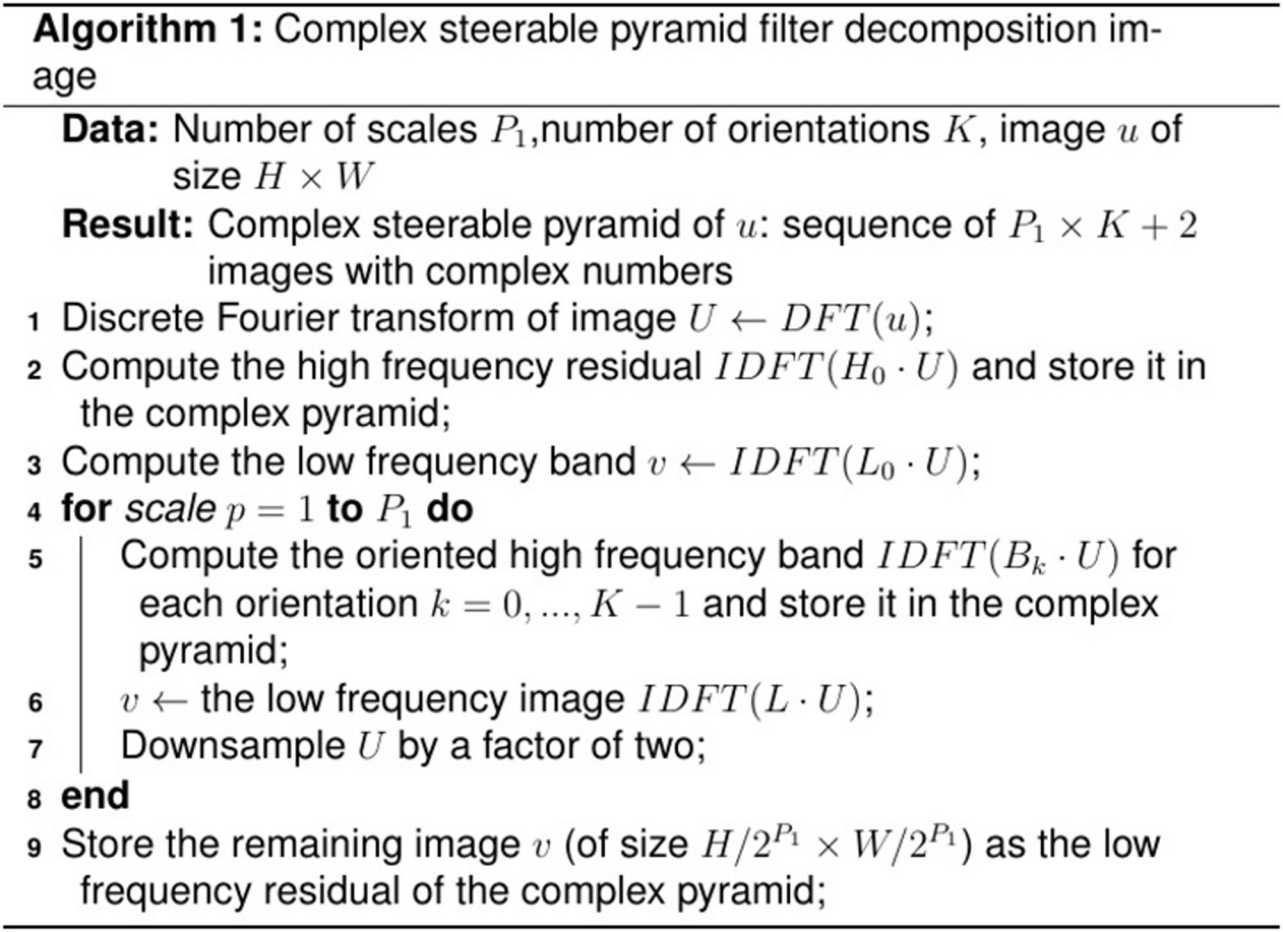
The pseudocode of the Octave CSP filter decomposition image algorithm.

Similar to the original Octave pyramid filter image decomposition algorithm [36], the principle of the Octave CSP filter image decomposition algorithm is to recursively multiply the high-pass filter *H*_0_ (*r*,*θ*), low-pass filter *L*_0_ (*r*,*θ*), and oriented band-pass filter *B*_*k*_ (*r*,*θ*) at different frequency domain scales with the image frequency domain. The corresponding time domain operation is shown in Eq (1), and the overall process is shown in Fig 3.

When Octave CSP is used for PBOF, its down-sampling rate can be set to 0 or 2. However, when Octave CSP is used for video motion amplification, as shown in Fig 3, its down-sampling rate can only be set to 2. In Fig 3, the number of orientations of the Octave CSP’s oriented band-pass filter *K* = 4 and the number of pre-decomposition scales *P*_1_ are calculated as:

$$P_{1}=\left[\log_{2}(min(W,H))\right]-2$$ (9)

where *min*(•) represents the minimum function; [•] is the integral function while *H* and *W* represent the length and width of images respectively. The calculation formulas of *H*_0_ (*r*,*θ*) and *L*_0_ (*r*,*θ*) are as follows:

$$H_{0}(r,\theta )=H\left(\frac{r}{2},\theta \right)$$ (10)

$$L_{0}(r,\theta )=L\left(\frac{r}{2},\theta \right)$$ (11)

where *r* and *θ* are the polar coordinates of the image frequency domain (*r*,*θ*), *r∈R*,*θ∈*(-π,π]. The calculation formulas of *H*(*r*,*θ*) and *L*(*r*,*θ*) are as follows:

$$H(r,\theta )=\left\{\begin{array}{c} 0,r\leq \frac{\pi }{4}\\ cos\left(\frac{\pi }{2}log_{2}\left(\frac{2r}{\pi }\right)\right),\frac{\pi }{4}<r<\frac{\pi }{2}\\ 1,r\geq \frac{\pi }{2} \end{array}\right.$$ (12)

$$L(r,\theta )=\left\{\begin{array}{c} 1,r\leq \frac{\pi }{4}\\ cos\left(\frac{\pi }{2}log_{2}\left(\frac{4r}{\pi }\right)\right),\frac{\pi }{4}<r<\frac{\pi }{2}\\ 0,r\geq \frac{\pi }{2} \end{array}\right.$$ (13)

The calculation formula of *B*_*k*_ (*r*,*θ*) is:

$$B_{k}(r,\theta )=H(r,\theta )G_{k}(\theta ),k\in [0,K-1]$$ (14)

$$G_{k}(\theta )=\left\{\begin{array}{c} \alpha_{K}{\left[cos\left(\theta -\frac{\pi k}{K}\right)\right]}^{K-1},|\theta -\frac{\pi k}{K}|<\frac{\pi }{2}\\ 0,otherwise \end{array}\right.$$ (15)

$$\alpha_{K}=2^{k-1}\frac{(k-1)!}{\sqrt{K[2(K-1)]!}}$$ (16)

In Eq (14), *H*(*r*,*θ*) and *G*_*k*_ (*θ*) respectively control the amplitude-frequency characteristics and rotation direction of the Octave CSP oriented band-pass filter.

#### 2.2.2 HalfOctave CSP

When HalfOctave CSP is used for PBOF, its down-sampling rate can be set to 0 or 1.41. In the HalfOctave CSP, the number of orientations *K* = 8; the number of pre-decomposition scales *P*_2_ = *P*_1_×2, and the radial bandwidths of the high-pass filter *H*_1_ (*r*,*θ*) and the low-pass filter *L*_1_ (*r*,*θ*) are 1/2 of Octave CSP’s *H*_0_ (*r*,*θ*) and *L*_0_ (*r*,*θ*). The calculation formulas are as follows:

$$H_{1}(r,\theta )=H_{11}\left(\frac{r}{2^{0.5}},\theta \right)$$ (17)

$$L_{1}(r,\theta )=L_{11}\left(\frac{r}{2^{0.5}},\theta \right)$$ (18)

Among them, the calculation formulas of *H*_11_ (*r*,*θ*) and *L*_11_ (*r*,*θ*) are as follows:

$$H_{11}(r,\theta )=\left\{\begin{array}{c} 0,r\leq \frac{\pi }{2}\\ cos\left(\pi log_{2}\left(\frac{2^{0.5}r}{\pi }\right)\right),\frac{\pi }{2}<r<\frac{\pi }{2^{0.5}}\\ 1,r\geq \frac{\pi }{2^{0.5}} \end{array}\right.$$ (19)

$$L_{11}(r,\theta )=\left\{\begin{array}{c} 1,r\leq \frac{\pi }{2}\\ cos\left(\pi log_{2}\left(\frac{2r}{\pi }\right)\right),\frac{\pi }{2}<r<\frac{\pi }{2^{0.5}}\\ 0,r\geq \frac{\pi }{2^{0.5}} \end{array}\right.$$ (20)

The differences in oriented band-pass filters between the HalfOctave CSP and the Octave CSP are shown in Fig 2(A) and 2(B). Specifically, the bandwidth and the number of orientations of the oriented band-pass filter *B*_*k*1_ (*r*,*θ*)of HalfOctave CSP are 1/2 and 2 times those of *B*_*k*_ (*r*,*θ*) of Octave CSP, respectively. The calculation formula of *B*_*k*1_ (*r*,*θ*) is:

$$B_{k1}(r,\theta )=H_{11}(r,\theta )G_{k}(\theta ),k\in [0,K-1]$$ (21)

Comparing Eqs (14) and (21), it can be seen that compared with the oriented band-pass filter of Octave CSP, the HalfOctave CSP oriented band-pass filter has a narrower bandwidth under the control of *H*_11_ (*r*,*θ*) and has more rotatable directions under the setting of the number of rotation directions *K*.

#### 2.2.3 SmoothHalfOctave CSP

When SmoothHalfOctave CSP is used for PBOF, its down-sampling rate can be set to 0 or 1.41. In the SmoothHalfOctave CSP, the number of orientations *K* = 8; the number of pre-decomposition scales *P*_3_ = *P*_1_×2, and the radial bandwidths of the high-pass filter *H*_2_ (*r*,*θ*) and the low-pass filter *L*_2_ (*r*,*θ*) are 1/2 of Octave CSP’s *H*_0_ (*r*,*θ*) and *L*_0_ (*r*,*θ*) with smoother amplitude-frequency characteristic. The calculation formulas are as follows:

$$H_{2}(r,\theta )=H_{21}\left(\frac{r}{2^{0.5}},\theta \right)$$ (22)

$$L_{2}(r,\theta )=L_{21}\left(\frac{r}{2^{0.5}},\theta \right)$$ (23)

The calculation formulas of *H*_21_ (*r*,*θ*) and *L*_21_ (*r*,*θ*) are as follows:

$$H_{21}(r,\theta )=\left\{\begin{array}{c} 0,r\leq \frac{\pi }{2}\\ 2^{6}\frac{6!}{\sqrt{7(2\times 6)!}}cos^{6}\left(\pi log_{2}\left(\frac{2^{0.5}r}{\pi }\right)\right),\frac{\pi }{2}<r<\frac{\pi }{2^{0.5}}\\ 1,r\geq \frac{\pi }{2^{0.5}} \end{array}\right.$$ (24)

$$L_{21}(r,\theta )=\left\{\begin{array}{c} 1,r\leq \frac{\pi }{2}\\ 2^{6}\frac{6!}{\sqrt{7(2\times 6)!}}cos^{6}\left(\pi log_{2}\left(\frac{2r}{\pi }\right)\right),\frac{\pi }{2}<r<\frac{\pi }{2^{0.5}}\\ 0,r\geq \frac{\pi }{2^{0.5}} \end{array}\right.$$ (25)

The differences in oriented band-pass filters between the SmoothHalfOctave CSP and the Octave CSP are shown in Fig 2(A) and 2(C). Specifically, the bandwidth and the number of orientations of the oriented band-pass filter *B*_*k*2_ (*r*,*θ*) of SmoothHalfOctave CSP are 1/2 and 2 times those of *B*_*k*_ (*r*,*θ*) of Octave CSP, respectively, and the amplitude-frequency characteristic of *B*_*k*2_ (*r*,*θ*) is smoother. The calculation formula of *B*_*k*2_ (*r*,*θ*) is:

$$B_{k2}(r,\theta )=H_{21}(r,\theta )G_{k}(\theta ),k\in [0,K-1]$$ (26)

Comparing Eqs (21) and (26), it can be seen that the oriented band-pass filter of the SmoothHalfOctave CSP has a smoother amplitude-frequency characteristic under the control of *H*_21_ (*r*,*θ*) compared to the oriented band-pass filter of the HalfOctave CSP.

#### 2.2.4 QuarterOctave CSP

When QuarterOctave CSP is used for PBOF, its down-sampling rate can be set to 0 or 1.19. In the QuarterOctave CSP, the number of orientations *K* = 8; the number of pre-decomposition scales *P*_4_ = *P*_1_×4, and the radial bandwidths of the high-pass filter *H*_3_ (*r*,*θ*) and the low-pass filter *H*_3_ (*r*,*θ*) are 1/4 of Octave CSP’s *H*_0_ (*r*,*θ*) and *L*_0_ (*r*,*θ*) with smoother amplitude-frequency characteristic. The calculation formulas are as follows:

$$H_{3}(r,\theta )=H_{31}\left(\frac{r}{2^{0.25}},\theta \right)$$ (27)

$$L_{3}(r,\theta )=L_{31}\left(\frac{r}{2^{0.25}},\theta \right)$$ (28)

where the calculation formulas of *H*_31_ (*r*,*θ*) and *L*_31_ (*r*,*θ*) are as follows:

$$H_{31}(r,\theta )=\left\{\begin{array}{c} 0,r\leq \frac{\pi }{2^{0.5}}\\ 2^{6}\frac{6!}{\sqrt{7(2\times 6)!}}cos^{6}\left(2\pi log_{2}\left(\frac{2^{0.25}r}{\pi }\right)\right),\frac{\pi }{2^{0.5}}<r<\frac{\pi }{2^{0.25}}\\ 1,r\geq \frac{\pi }{2^{0.25}} \end{array}\right.$$ (29)

$$L_{31}(r,\theta )=\left\{\begin{array}{c} 1,r\leq \frac{\pi }{2^{0.5}}\\ 2^{6}\frac{6!}{\sqrt{7(2\times 6)!}}cos^{6}\left(2\pi log_{2}\left(\frac{2^{0.5}r}{\pi }\right)\right),\frac{\pi }{2^{0.5}}<r<\frac{\pi }{2^{0.25}}\\ 0,r\geq \frac{\pi }{2^{0.25}} \end{array}\right.$$ (30)

The differences in oriented band-pass filters between the QuarterOctave CSP and the Octave CSP are shown in Fig 2(A) and 2(D). Specifically, the bandwidth and the number of orientations of the oriented band-pass filter *B*_*k*3_ (*r*,*θ*) of QuarterOctave CSP are 1/4 and 2 times those of *B*_*k*_ (*r*,_*θ*_) of Octave CSP, respectively, and the amplitude-frequency characteristic of *B*_*k*3_ (*r*,*θ*) is smoother. The calculation formula of *B*_*k*3_ (*r*,*θ*) is:

$$B_{k3}(r,\theta )=H_{31}(r,\theta )G_{k}(\theta ),k\in [0,K-1]$$ (31)

Comparing Eqs (26) and (31), it can be seen that the oriented band-pass filter of QuarterOctave CSP has a narrower bandwidth under the control of *H*_31_ (*r*,*θ*) compared to the oriented band-pass filter of SmoothHalfOctave CSP.

## 3 Methodology

**Fig 4:**
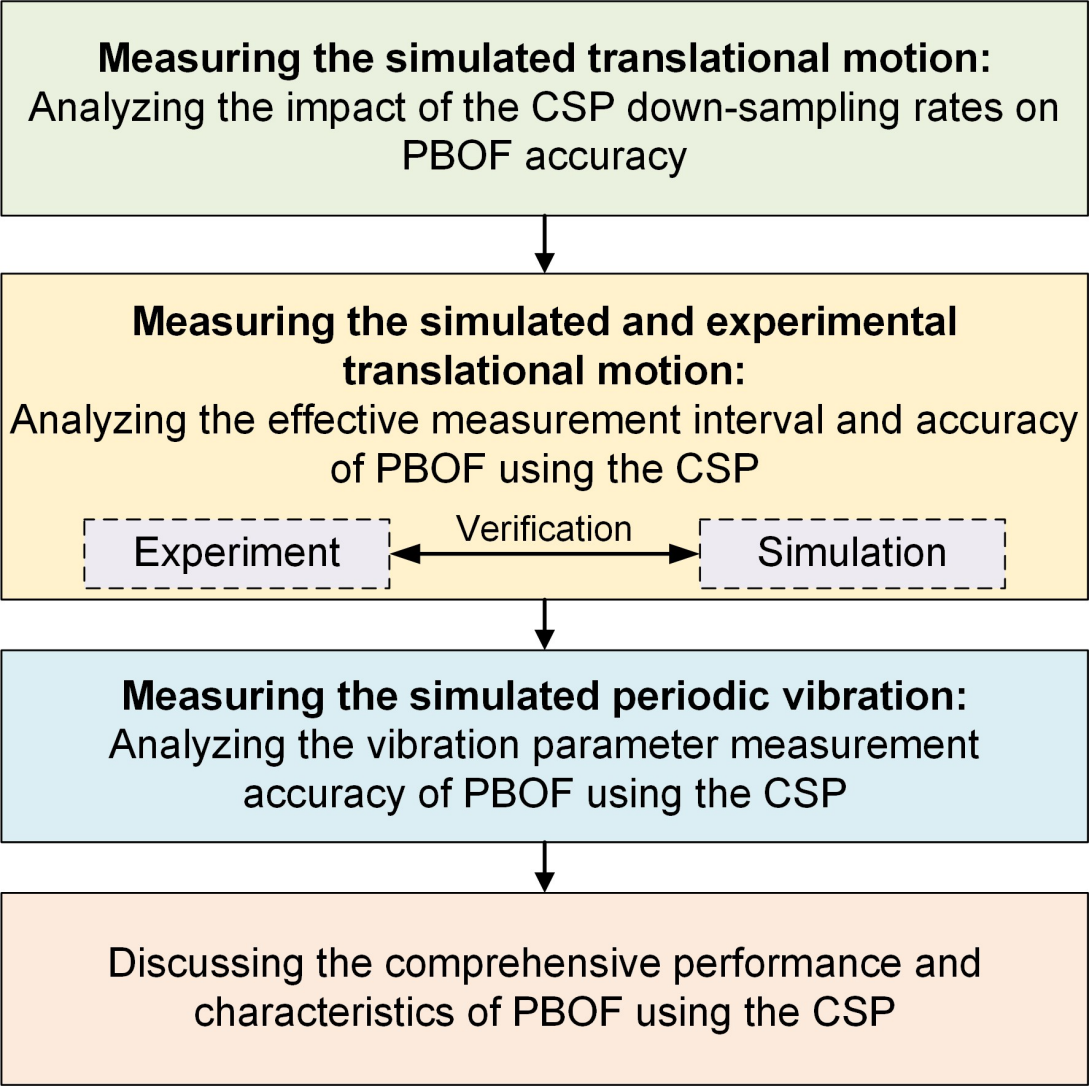
Performance evaluation flow chart of motion parameter measurement with PBOF using four types of CSP.

In this study, the impact of different CSP filter types (Octave, HalfOctave, SmoothHalfOctave, and QuarterOctave) and parameters on the performance of PBOF in measuring motion parameters is evaluated using simulations and experiments. The process is shown in Fig 4. Firstly, through measuring the simulated translational motion, the influence of CSP’s down-sampling rates on the displacement measurement accuracy of the PBOF is analyzed to select the appropriate settings for subsequent research. Next, the effective displacement measurement interval and accuracy of PBOF using the CSP are studied by measuring the simulated and experimental translational motion. Then, the vibration parameter measurement accuracy of PBOF using the CSP is analyzed by measuring the simulated periodic vibration. Finally, the comprehensive performance and characteristics of PBOF using the CSP are discussed.

### 3.1 Performance evaluation metrics

In the simulation and experiment of translational motion measurement (section 3.2), the relative error (RE) and absolute error (AE) are employed to assess the accuracy of the final measured displacement ***m*** obtained by PBOF using a CSP filter on a per-frame basis. The calculation formulas for RE and AE are as follows:

$$RE=\frac{\left|m-r\right|}{\left|r\right|}\times 100\text{\%}$$ (32)

AE=|m−r| (33)

In the formula, *r* represents the true displacement of a single frame image. The RE metric can simultaneously reflect the errors at different scales of displacement, while the AE metric intuitively reflects the magnitude of measurement errors. The effective displacement measurement interval described in this paper pertains to the interval of displacements where the RE is less than 10%.

Time-domain and frequency-domain evaluation metrics are used to assess the overall accuracy of the measured vibration displacement curves (section 3.3). The time-domain evaluation metrics include the correlation coefficient *R*^2^ between measured and actual vibration displacement, as well as the vibration amplitude absolute error (VAAE) and the root mean square error (RMSE). The frequency-domain evaluation metric uses the maximum frequency amplitude absolute error (MFABE) to quantify the absolute error between the measured and actual vibration frequencies. The calculation formulas for RMSE, VAAE, *R*^2^, and MFABE are as follows:

$$RMSE=\sqrt{\frac{1}{N}\sum_{i=1}^{N}{\left(m_{i}-r_{i}\right)}^{2}}$$ (34)

In the formula, *m*_*i*_ is the displacement measurement of the *i*-th frame; *r*_*i*_ is the true displacement of the *i*-th frame; *N* is the number of all measurement frames.


$$VAAE=|m_{max}-r_{max}|$$ (35)

In the formula, *m*_*max*_ is the maximum measured displacement over *N* frames; *r*_*max*_ is the maximum true displacement over *N* frames.


$$R^{2}=\frac{1}{N-1}\sum_{I=1}^{N}\left(\frac{m_{i}-u_{m}}{\sigma_{m}}\right)\left(\frac{r_{i}-u_{r}}{\sigma_{r}}\right)$$ (36)

In the formula, *u*_*m*_ is the mean of the measured displacements over *N* frames, σ_*m*_ is the standard deviation of the measured displacements over *N* frames; *u*_*r*_ is the mean of the true displacements over *N* frames, and σ_*r*_ is the standard deviation of the true displacements over *N* frames.


$$MFABE={\left.||P\left(f_{M}\right)\right|}_{max}-{\left|P\left(f_{R}\right)\right|}_{max}|$$ (37)

In the formula, |*P*(*f*_*M*_)| and |*P*(*f*_*R*_)| respectively represent the frequency magnitude of the measured displacement and the frequency magnitude of the true displacement.

### 3.2 Measurement of translational motion of simulation and experiment

#### 3.2.1 Analysis of the influence of down-sampling rates of the CSP

**Fig 5:**
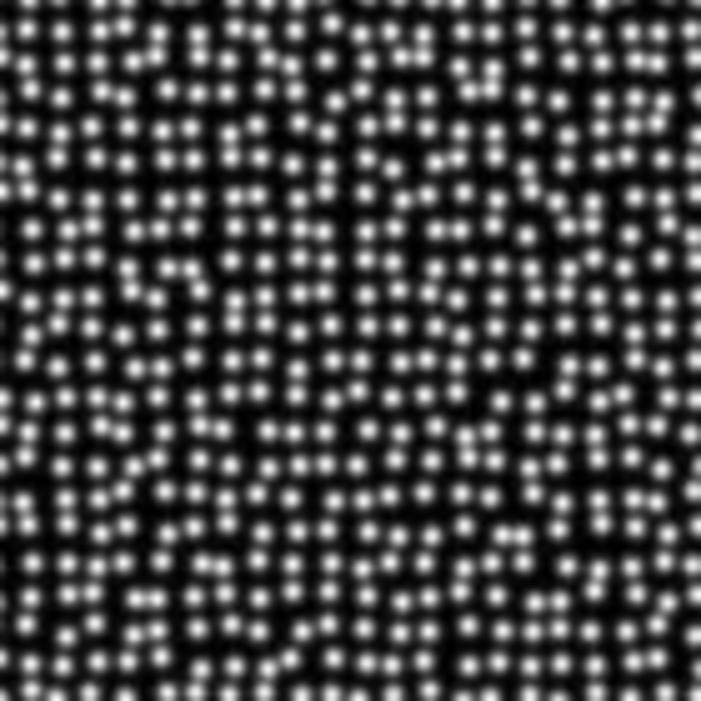
Simulated speckle reference image.

In this section, a simulation speckle reference image is generated with a resolution of 140×140 pixels, as depicted in Fig 5, following the recommended settings of the Glare algorithm [37]. Then, the Binning method [38] is applied with a step size of 0.1 pixel, consecutively shifting the speckle reference image along the positive *x* direction ten times. This process creates displacements ranging from 0 to 1 pixel.

The down-sampling rate of the CSP, which affects the grayscale noise intensity of images, the spatial reach of the CSP filter, and the dimensions of the output spatial domain response map at each CSP filter level, significantly influences the measurement accuracy of PBOF. Thus, applying optimal CSP filters with varying down-sampling rates is employed to measure the displacement of speckle image sequences through PBOF. The influence of down-sampling rates on PBOF’s displacement measurement accuracy is analyzed to determine appropriate settings for subsequent investigations by comparing the average RE and average AE.

#### 3.2.2 Analysis of effective displacement measurement interval and accuracy

**Fig 6:**
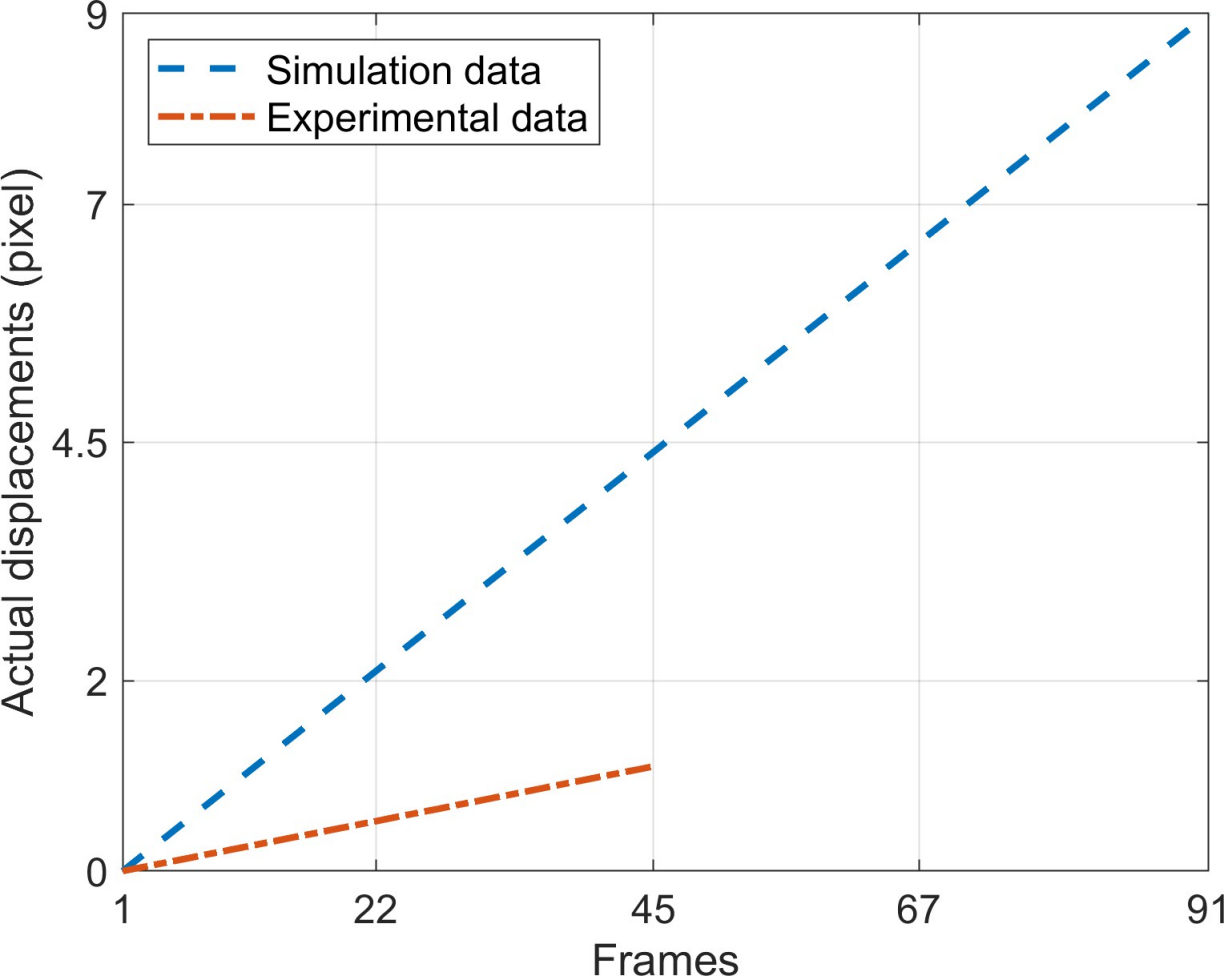
The *x* direction displacements provided by the data in Section 3.2.2.

**Fig 7:**
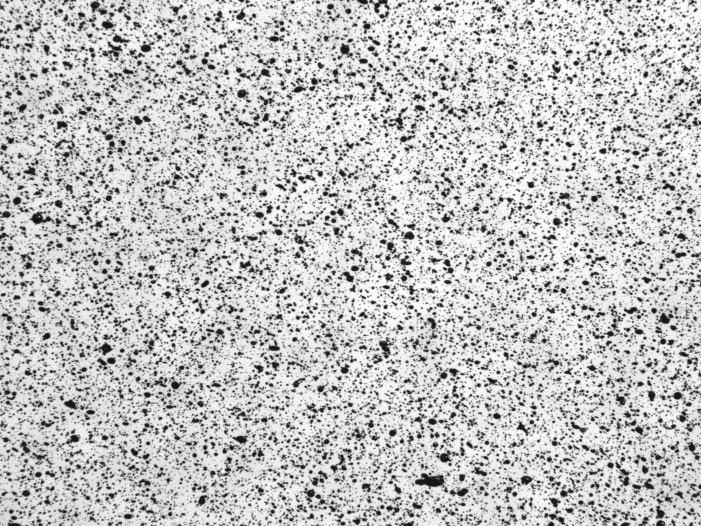
Experimental speckle reference image.

The data used in this section comes from two parts: the simulated data generated in this section and the experimental data shared in reference [39]; they provide displacements as depicted in Fig 6. The experimental data contains 45 consecutive sub-pixel rigid body translation speckle images with an image size of 1600×1200 pixels and an exposure time of 1000us, its reference speckle image is depicted in Fig 7. Since the experimental data can provide a maximum displacement of 1.1 pixel, assessing the maximum effective measurement displacement of PBOF using the CSP is impossible. Therefore, the maximum displacement in the simulated data is set to 9 pixels, more significant than the upper displacement limit in most applications. Additionally, the size of the simulated speckle reference image is consistent with the experimental data, and the remaining configurations are the same as those in section 3.2.1.

Firstly, at the optimal down-sampling rate, the displacement of image sequences in the simulation is measured by PBOF using CSP, and RE and AE are calculated for each frame. According to the evaluation criteria in Section 3.1, the effective displacement measurement interval is analyzed. Then, based on the average value of RE within both the micro-displacement interval (0~1 pixels) and the effective measurement interval, the measurement accuracy of PBOF using CSP within these two ranges is analyzed.

Subsequently, applying the same methodology as described above, an analysis is performed on the minimum effective measurement displacement and the accuracy within the range of 0 to 1.1 pixels using experimental data. Finally, the experimental results are compared with the simulation results in this section.

### 3.3 Measurement of parameters of simulation vibration

In applying vibration displacement measurement and structural modal analysis, more attention is paid to the variation trend of displacement measurement and the accuracy of frequency domain parameter measurement instead of the accuracy of displacement measurement itself. Therefore, the purpose of the simulation in this section is to analyze the accuracy of PBOF using the CSP to measure vibration parameters.

**Fig 8:**
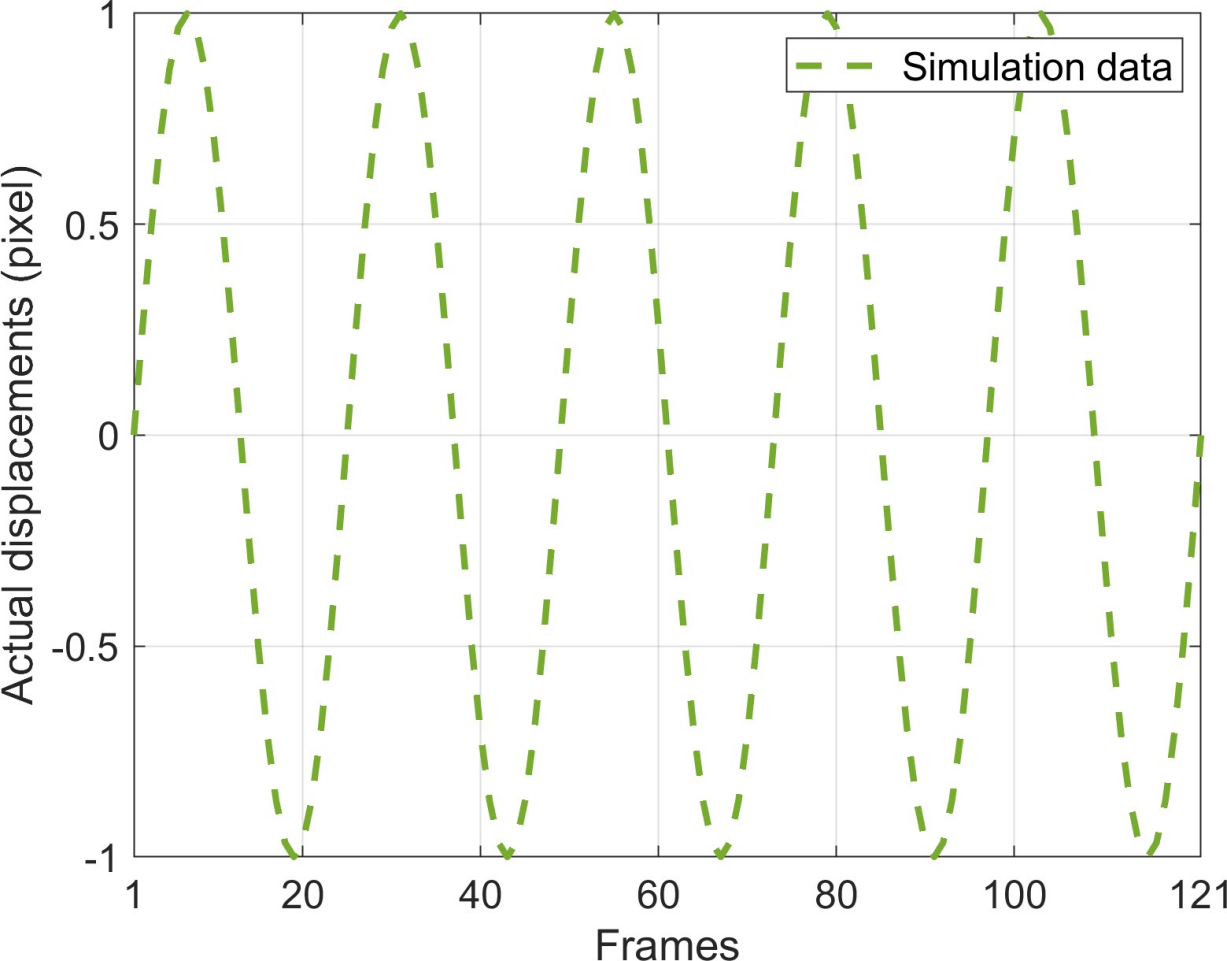
The x direction displacements provided by the data in Section 3.3.

In this section, the simulated speckle reference image in Section 3.2.2 undergoes sinusoidal vibration displacement with an amplitude of 1 pixel along the x direction, a vibration frequency of 5 Hz, a sampling frequency of 120 Hz, and a sampling time of 1 second, respectively. One hundred twenty frames of vibration image sequences are generated, and the corresponding provided displacements are illustrated in Fig 8.

After the vibration image sequence is generated, the displacement of the vibration image sequence is measured by PBOF using the CSP at the optimal down-sampling rate. Then, the calculations for *R*^2^, VAAE, RMSE, and MFABE between the measured vibration displacement and the actual vibration displacement are conducted, and the accuracy of the PBOF the CSP to measure the vibration parameters is analyzed.

## 4 Results

### 4.1 Hardware and software platform and pyramid decomposition scale

The hardware platform configuration that runs the simulation and experimental program in this section is dual Intel (R) Xeon (R) Gold 6139M CPU, dual Nvidia GTX3080 GPU, and 128GB memory. Under the Windows 10 operating system, the software algorithm is implemented using the Matlab 2021 (a) platform.

When measuring the displacement of the simulated and experimental image sequence with PBOF using four types of CSP, each CSP has multiple pre-decomposition scales, and different pre-decomposition scales can independently obtain a motion parameter measurement result in different directions. Since the displacement of the simulated and experimental image sequences in this paper is along the *x* direction, the oriented band-pass filter with a rotation angle of 0 when pyramids are on different pre-decomposition scales is selected to measure the displacement. Therefore, *P*_1_, *P*_2_, *P*_3_, and *P*_4_ different measurement results can be obtained respectively with PBOF using Octave, HalfOctave, SmoothHalfOctave, and QuarterOctave. According to the introduction of the calculation method of the decomposition layers of CSP in Section 2.2, for the image data provided in Section 3.2.1, the decomposition layers of each type of CSP are: *P*_1_ = 5, *P*_2_ = *P*_3_ = 10, and *P*_4_ = 20; for the image data provided in Sections 3.2.2 and 3.3, the decomposition layers of each type of CSP are: *P*_1_ = 8, *P*_2_ = *P*_3_ = 16, and *P*_4_ = 32, respectively.

According to the introduction in Section 2.2 regarding the down-sampling rates that can be set for CSP when applied to PBOF, the optional down-sampling rates *d* for Octave, HalfOctave, SmoothHalfOctave, and QuarterOctave CSP are 2 and 0, 1.41 and 0, 1.41 and 0, 1.19 and 0, respectively. When *d* is not equal to 0, the spatial domain response image size of the oriented band-pass filter at the *l*^th^ level is 1/*d* as large as that of the oriented band-pass filter at the *l*-1^th^ level. The higher the level of the oriented band-pass filter is, the lower the spatial resolution of the spatial domain response image is. When *d* is equal to 0, the spatial resolution of the spatial domain response image of the oriented band-pass filter at each level is always the same as that of the original image.

### 4.2 Analysis of the influence of down-sampling rates of CSP

**Fig 9:**
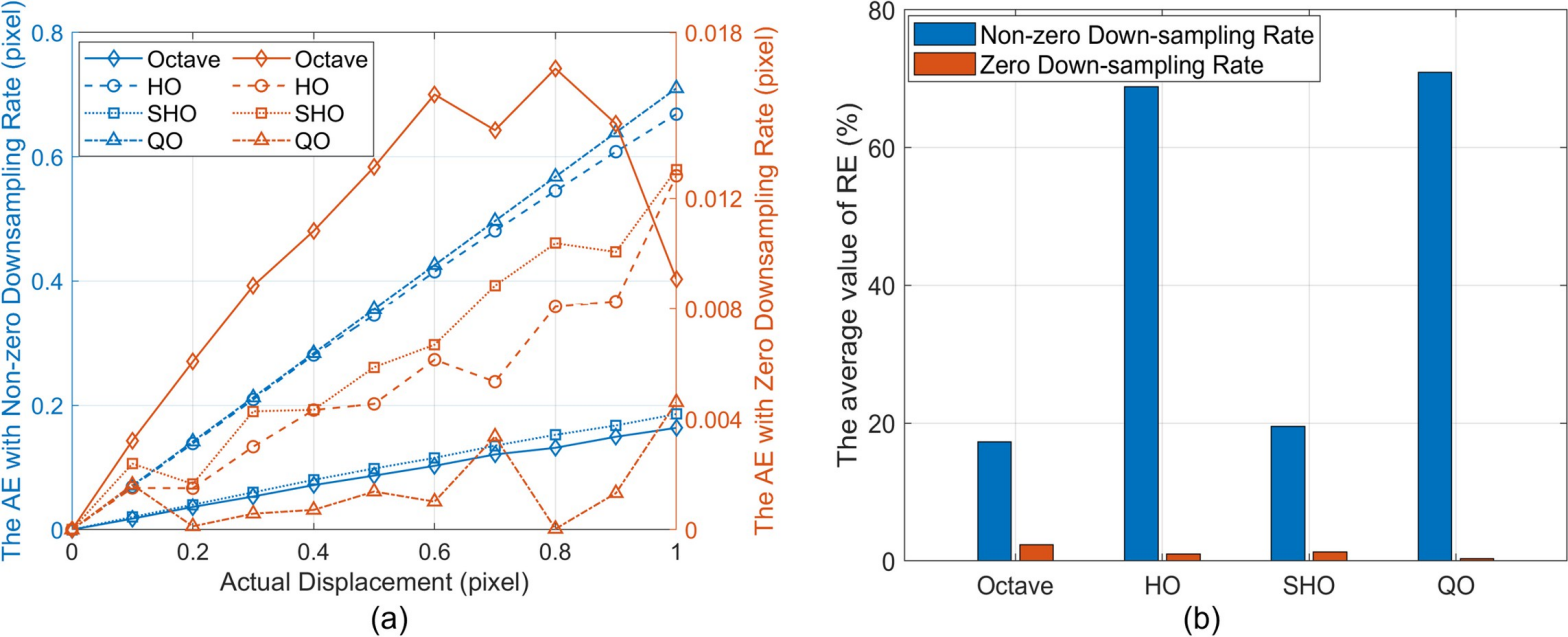
The displacement measurement accuracy of PBOF using the most accurate filters of CSP under various downsampling rates. (a) The value of AE across all frames. (b) The average value of RE across all frames. HO, SHO, and QO respectively represent HalfOctave, SmoothHalfOctave, and QuarterOctave.

When employing the simulated data from Section 3.2.1, the AE and the average value of RE across all frames obtained by PBOF using the most accurate filters from each CSP under various down-sampling rates are illustrated in Fig 9. According to the introduction of the optional down-sampling rate of CSP in Section 4.1, the non-zero down-sampling rates of Octave, HalfOctave, SmoothHalfOctave, and QuarterOctave CSP in Fig 9 are 2, 1.41, 1.41, and 1.19, respectively.

As shown in Fig 9(A), when the down-sampling rate of CSP is zero, the value of AE is reduced by over five times compared to when the down-sampling rate is non-zero. Simultaneously, in Fig 9(B), the average value of RE is reduced by over seven times. It is evident that with a down-sampling rate of zero, the displacement measurement accuracy of PBOF is significantly higher than when the rate is non-zero. This is because, according to the principle of CSP filter decomposition of images, when the down-sampling ratio of CSP is not zero, as the filter level increases, the resolution of the measured image will decrease, which will have an adverse effect on the displacement measurement performance of PBOF [32], whereas when the down-sampling ratio of CSP is zero, the increase in the filter level will not affect the edge resolution, in which case, the displacement measurement performance of PBOF is maximized [24].

In summary, because each category of CSP has only one non-zero down-sampling rate, and the measurement accuracy of PBOF is significantly higher when the down-sampling rate is zero compared to when it is non-zero, the down-sampling rates for CSP used in subsequent analyses are all set to zero.

### 4.3 Analysis of effective displacement measurement interval and accuracy

#### 4.3.1 Simulation results

**Fig 10:**
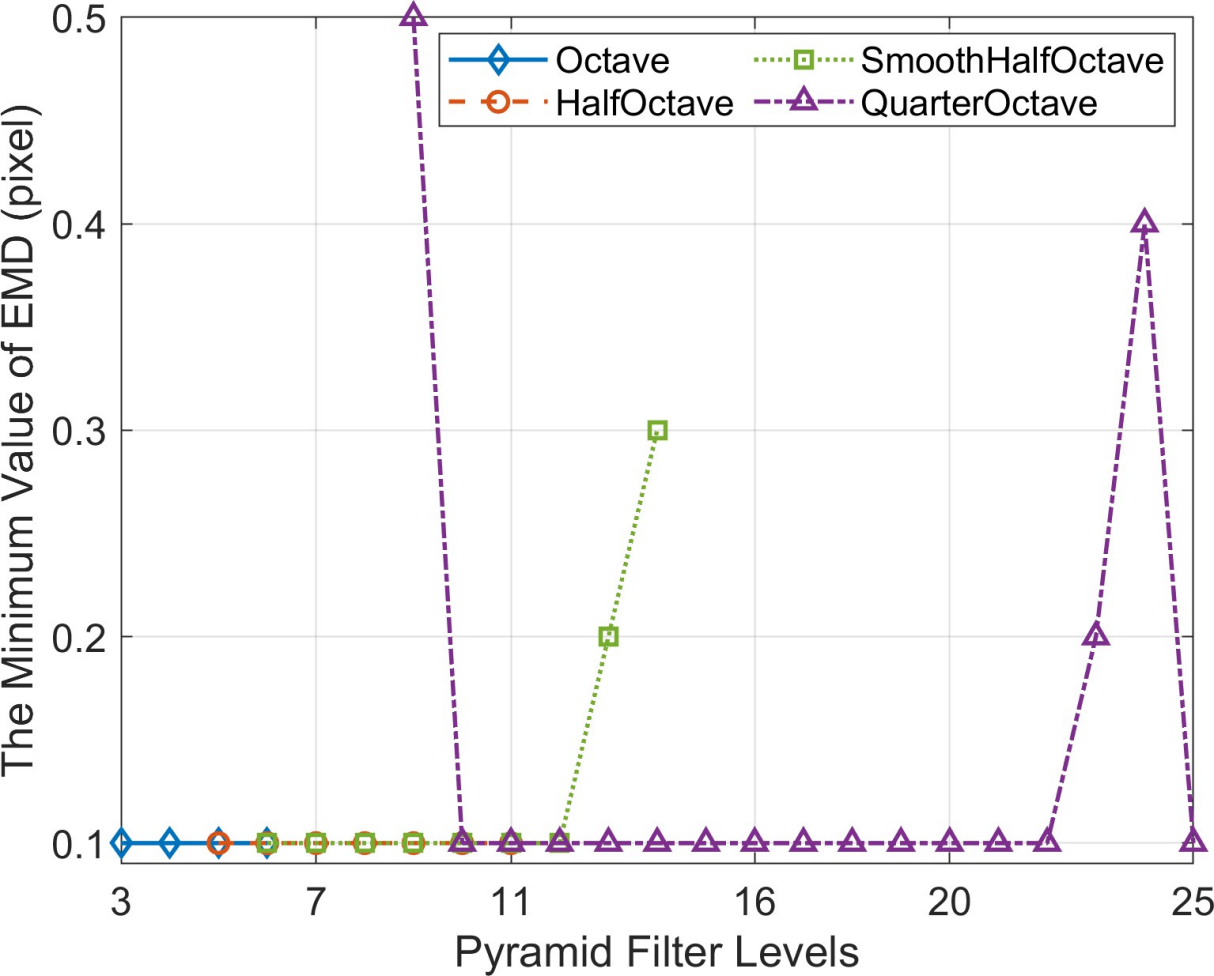
The minimum value of effective measurement displacement (EMD) obtained by PBOF using the effective level filters of each CSP.

When employing the simulated data described in Section 3.2.2 and applying the evaluation criteria for measurement validity described in Section 3.1, the minimum value of effective measurement displacement (EMD) results obtained by PBOF using the effective level filters of each CSP are presented in Fig 10. Most of each CSP’s effective level filters can achieve effective measurements of the 0.1-pixel micro-displacement simulated in the simulation. However, the micro-displacement sensing capability is limited when using the 13th and 14th level filters of the SmoothHalfOctave CSP, as well as the 9th, 23rd, and 24th level filters of the QuarterOctave CSP.

**Fig 11:**
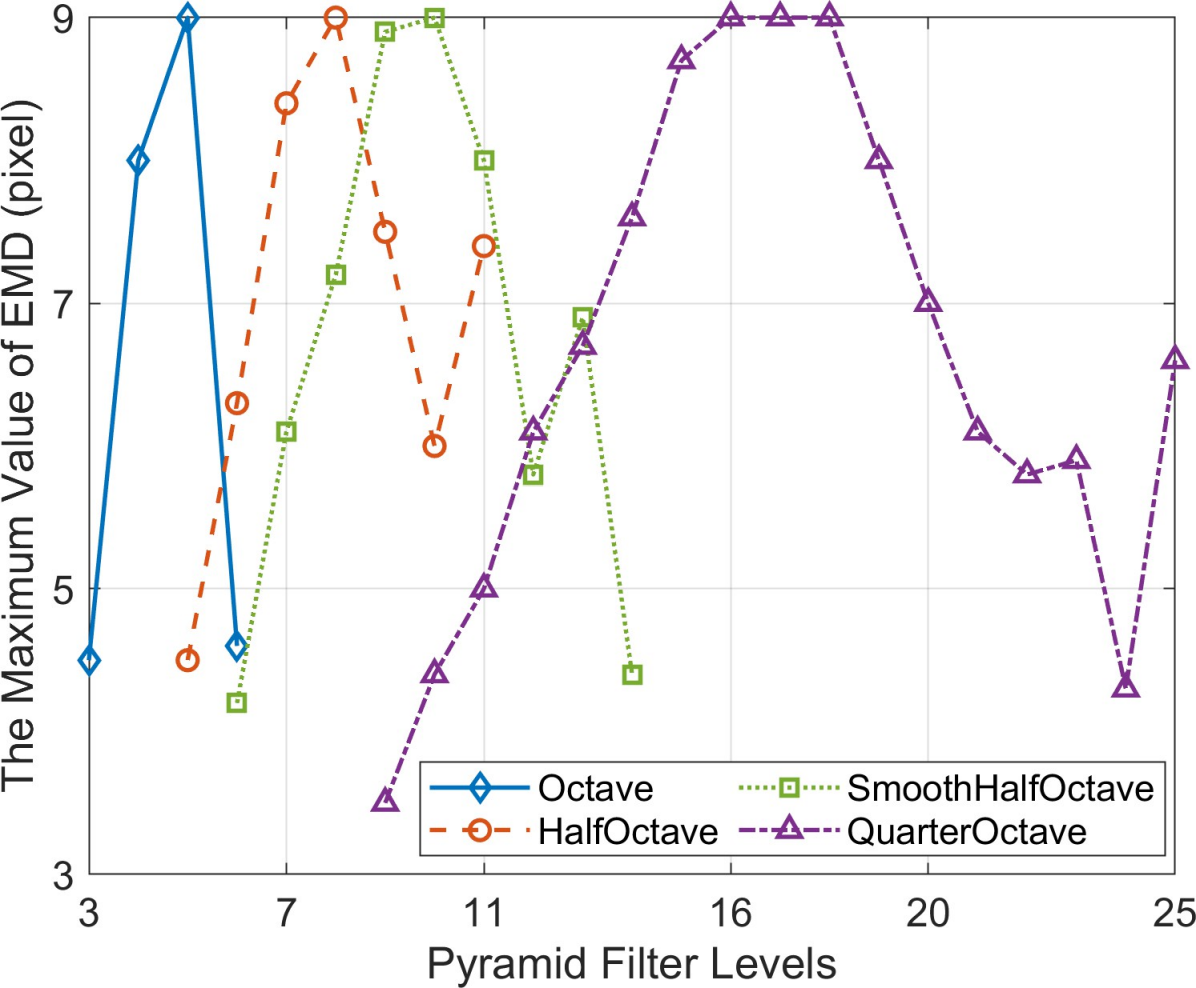
The maximum value of effective measurement displacement (EMD) obtained by PBOF using the effective level filters of each CSP.

**Fig 12:**
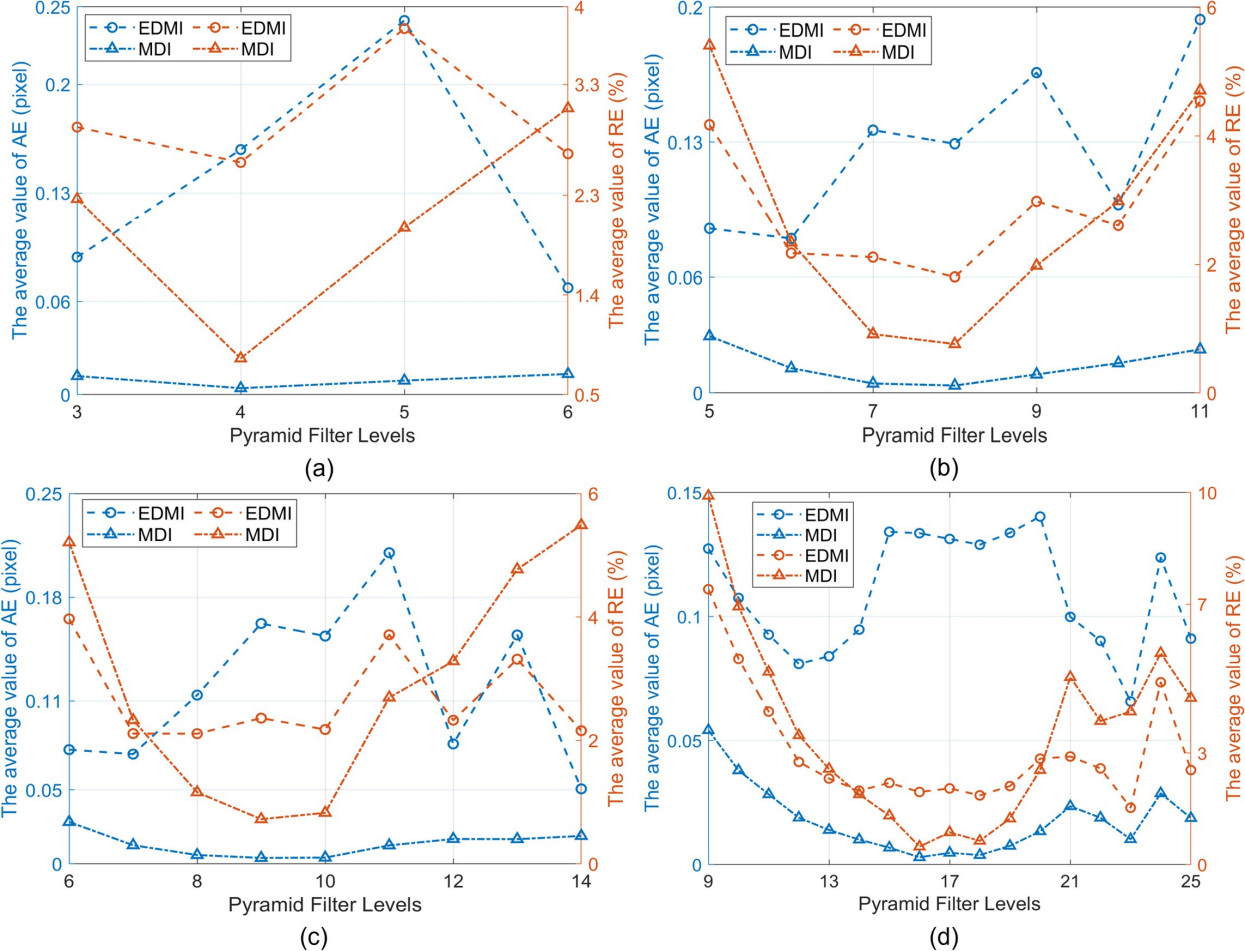
Within the micro-displacement interval (MDI) and the effective displacement measurement interval (EDMI), the average value of AE and the average value of RE of PBOF across all measurement frames using the effective level filters of CSP. (a) Octave. (b) HalfOctave. (c) SmoothHalfOctave. (d) QuarterOctave.

When using the effective level filters of CSP, the maximum value of EMD obtained by PBOF is shown in Fig 11. Within the micro-displacement interval (MDI) and the effective displacement measurement interval (EDMI), the average value of AE and the average value of RE of PBOF across all measurement frames using the effective level filters of CSP are shown in Fig 12. In Fig 11, it can be seen that no matter which CSP is used, the maximum value of EMD first increases with the increase of the filter level, and the displacement measurement ability is the strongest at about 1/2 of the effective filter level range of the CSP, which can realize the effective measurement of the maximum displacement (9 pixel) that can be simulated in the simulation. After that, the maximum value of EMD decreases with the increase of filter level.

It can also be seen from Fig 12 that the variation trend of the average value of RE corresponding to each CSP is almost the same as that of the maximum value of EMD. In other words, when employing the relatively middle-level filters of each CSP, PBOF demonstrates higher accuracy. Hence, when using the middle-level filter of each CSP, PBOF exhibits the most considerable displacement measurement interval, the highest measurement accuracy, and the best performance.

The premise for accurate measurement by PBOF with a CSP filter is to ensure that the motion phases obtained are not distorted [34]. As shown in Fig 10, when the level 13 and 14 filters of SmoothHalfOctave CSP and the level 9, 23, and 24 filters of QuarterOctave CSP are used, their micro-displacement perception ability is poor. The reason is that when the displacement of the image is smaller than the minimum effectively measurable displacement perceivable by the above filters, they are not able to obtain the accurate motion phase. Fig 11 shows that when the level 5 filter of Octave CSP, the level 8 filter of HalfOctave CSP, the level 10 filter of SmoothHalfOctave CSP, and the level 16–18 filters of QuarterOctave CSP are used, the maximum effectively measurable displacement of PBOF is 9 pixels. Analogously, this is because when the displacement of the image is 9 pixels, the above filters are still able to accurately obtain the motion phase. It is shown in Fig 12 that in the case of various CSPs, when filters located about 1/2 of the range of effective levels are used, PBOF is able to take measurements with higher accuracy in both the range of effectively measurable displacement and the range of micro-displacement measurement. This is because the filters at about 1/2 of the range of effective levels of various CSPs are able to obtain the motion phases more accurately in both ranges of measurable displacement.

**Fig 13:**
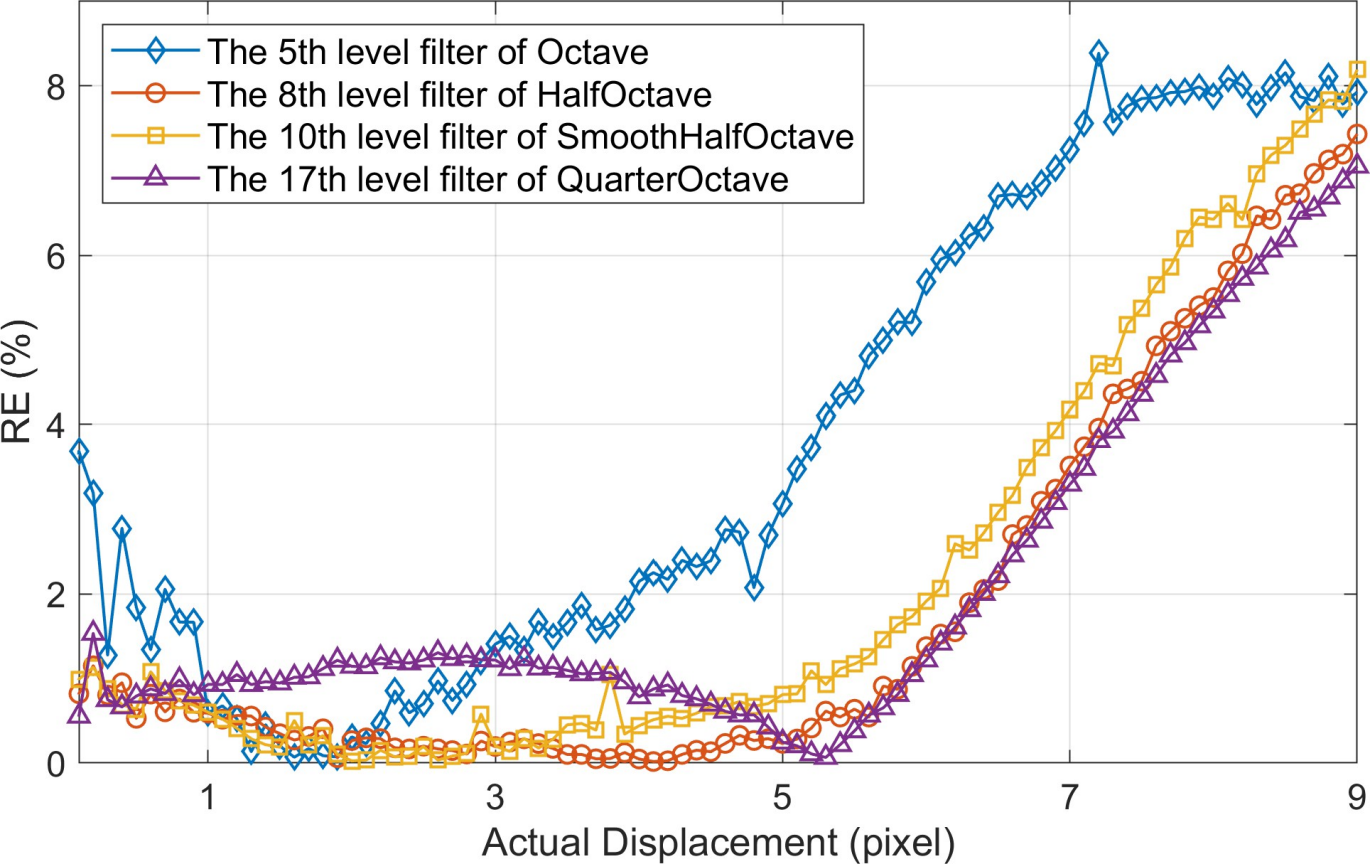
The RE of each frame image is measured in the simulated image sequence by PBOF using the most accurate filter of each CSP.

When using the most accurate filters of Octave, HalfOctave, SmoothHalfOctave, and QuarterOctave CSP respectively, the RE of each frame within the effective measurement range obtained by PBOF is shown in Fig 13. The values on the vertical axis of Fig 13 represent the relative error of PBOF when measuring a single frame image. In the 0~5 pixels displacement interval, the values of RE corresponding to the four types of CSP are less than 2%, and the single-frame measurement accuracy is relatively high. However, in the 5~9 pixels displacement interval, the single-frame measurement accuracy corresponding to each CSP decreases rapidly with the displacement increase, with a total decrease of about 6%. It can be seen from Fig 13 that no matter which CSP filter is used, under the simulation conditions herein, the displacement accuracy of PBOF will be significantly reduced when the displacement is greater than 5 pixels. This is because an important premise for accurate measurement by PBOF is that the small motion assumption can be established, allowing Eq (2) to be approximately expanded using the first-order Taylor formula [40]. When the displacement is larger, the small motion assumption can no longer be established, in which case it is impossible to guarantee the PBOF accuracy.

Furthermore, as indicated by Fig 13, the variation trend of RE of the single-frame displacement measurement obtained by the most accurate filter of each CSP is almost the same. However, in different displacement intervals, according to the average value of RE corresponding to each CSP, the difference in accuracy between the CSP with the highest accuracy and that with the lowest accuracy is relatively significant. Therefore, when the displacement interval changes, the characteristics of each CSP can be fully utilized to select the appropriate one according to the displacement interval to improve the accuracy.

#### 4.3.2 Experimental results

**Fig 14:**
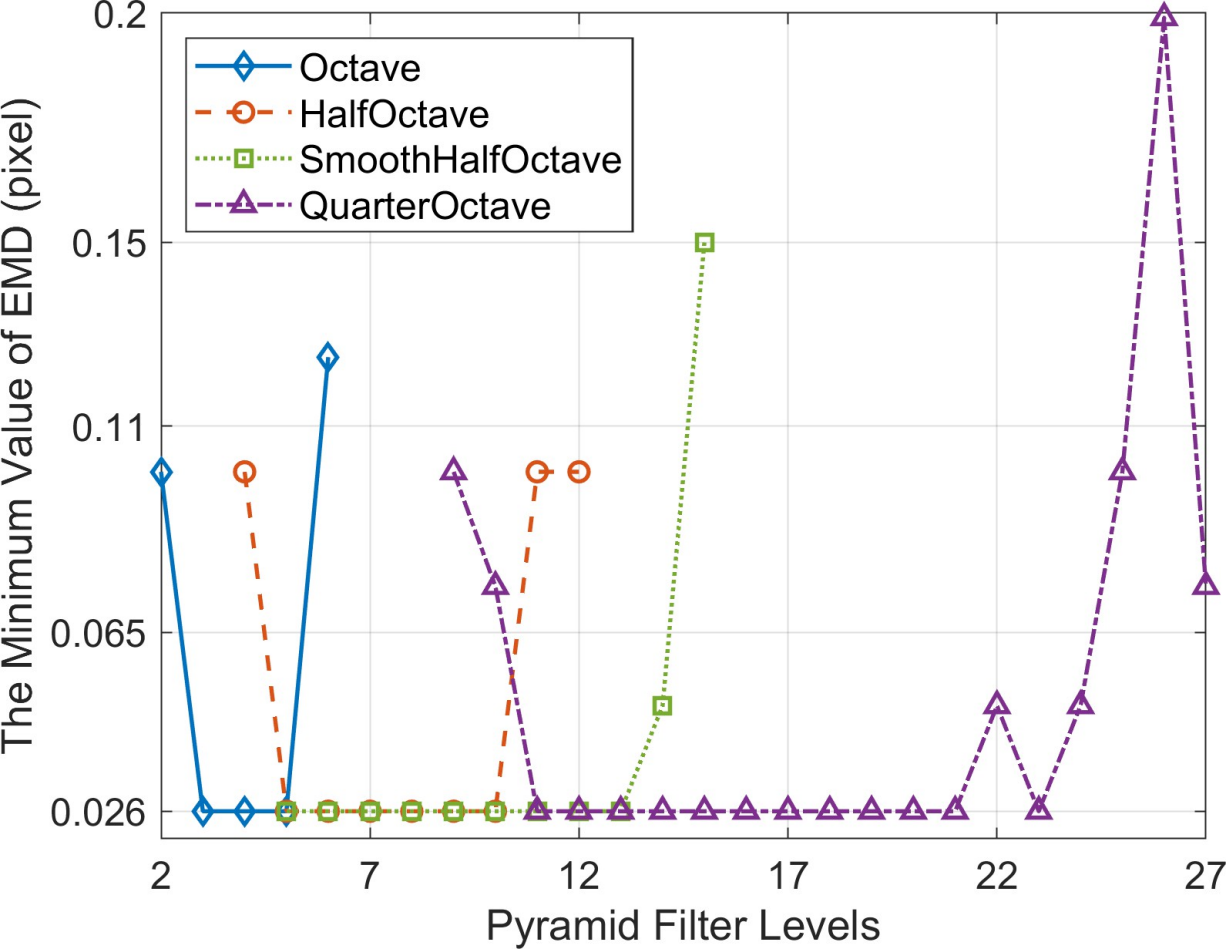
The minimum value of EMD obtained by PBOF using the effective level filters of each of CSP.

When utilizing the experimental data described in Section 3.2.2, the minimum value of EMD obtained by PBOF using the effective level filters of the four types of CSP is shown in Fig 14. It can be observed that PBOF using the most level filters of the four types of CSP can achieve effective measurements of the minimum displacement (0.026 pixel) provided by the experiment. However, for the 2nd and 6th level filters of the Octave CSP, the 4th, 11th, and 12th level filters of the HalfOctave CSP, the 14th and 15th level filters of the SmoothHalfOctave CSP as well as the 9th~10th, 22nd, and 24th~27th level filters of the QuarterOctave CSP, the micro-displacement sensing ability is limited.

**Fig 15:**
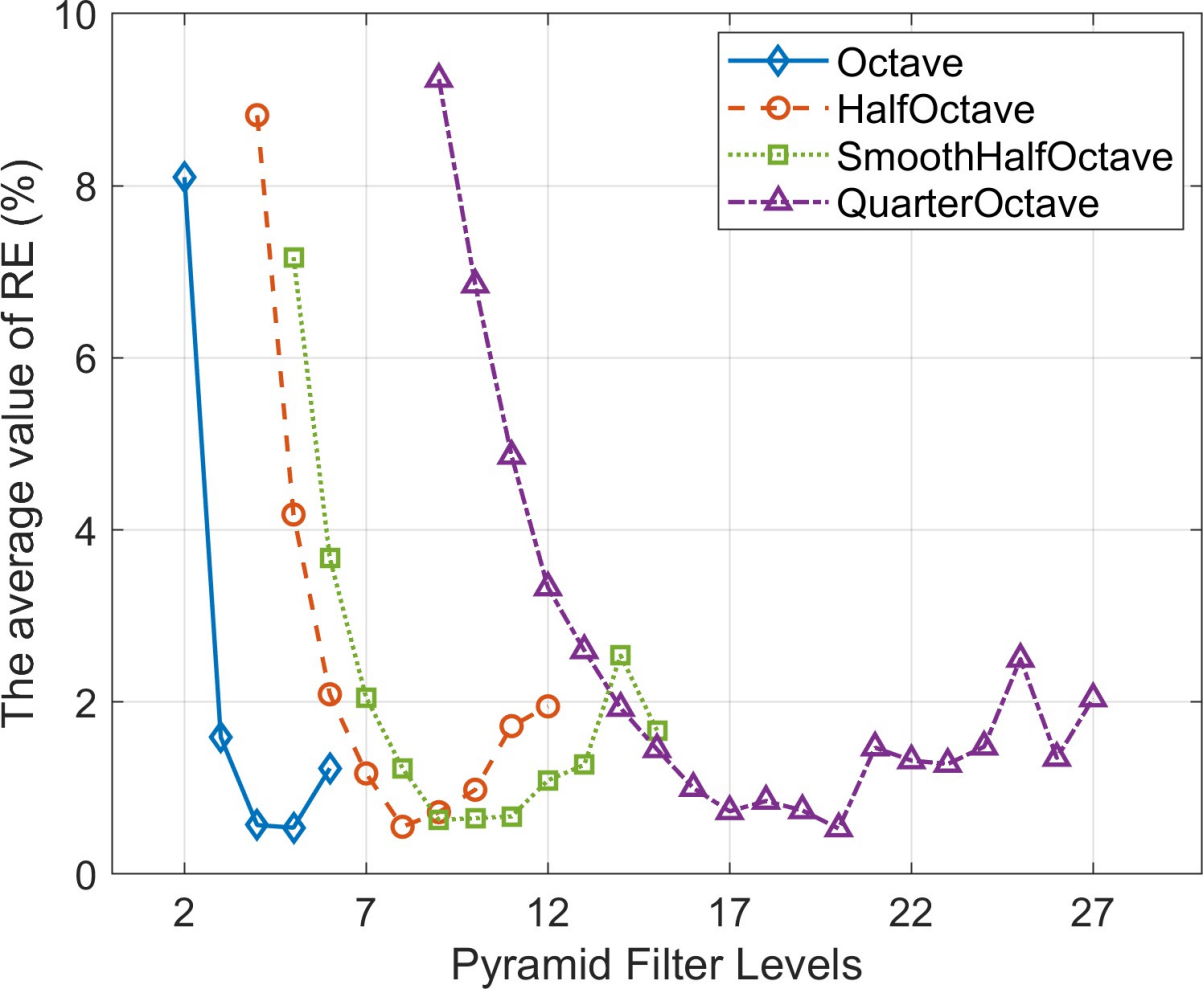
The average value of RE obtained by PBOF using the effective level filters of each CSP.

In the displacement interval provided by the experiment, the average value of RE obtained by PBOF using the effective level filters of the four types of CSP is shown in Fig 15. It can be seen from Fig 15 that no matter which CSP is used, the average value of RE decreases sharply with the increase of the filter level. The micro-displacement measurement accuracy is the highest at about 1/2 of each CSP’s effective filter level range. After that, the average value of RE gradually increases with the increment of filter levels. The filter level has a significant effect on the accuracy of micro-displacement measurement. When using the middle-level filter of each CSP, PBOF has the highest measurement accuracy.

The simulation results in Section 4.3.1 are highly consistent with the experimental results in this section. It can be concluded that: (I) PBOF using effective level filters of each CSP demonstrates a strong capability for perceiving micro-displacements. When using the QuarterOctave CSP, the largest number of filters with strong displacement perception ability can be obtained. (II) When PBOF uses the different effective level filters of the same CSP, the measurement accuracy will change significantly, and the highest measurement accuracy can be obtained when the middle-level filter is used.

### 4.4 Vibration parameter measurement results

When employing the effective level filters of each CSP, the error metric results for VAAE, RMSE, and MFABE obtained from PBOF measurements of displacement in the simulated periodic vibration motion data of Section 3.3 are presented in Table 1. Notably, in the MFABE metric, measured and actual vibrations’ maximum frequency amplitude values are at 5 Hz. Additionally, all effectively measured displacements exhibit a correlation coefficient (*R*^2^) of 1 with the actual displacements. It can be seen from Table 1 that no matter which CSP is used, the three kinds of measurement errors first decrease with the increase of the filter level. The measurement accuracy of the vibration parameters is the highest at about 1/2 of the effective filter level range of the CSP. Then, the three kinds of measurement errors increase with the increase of filter level. These rules are almost consistent with those manifested in the simulation and experiment of displacement measurement in Section 4.3.

**Table 1:** The VAAE (pixel), RMSE (pixel), and MFABE (Hz) of the measured vibration displacement obtained by PBOF using the effective level filters of each CSP.

Pyramid Type	Filter Level	VAAE	RMSE	MFABE	Pyramid Type	Filter Level	VAAE	NRMSE	MFABE
Octave	3	0.019	0.015	0.018	Smooth-HalfOctave	14	0.018	0.088	0.062
	4	0.006	0.005	0.006					
	5	0.006	0.017	0.019	Quartr-Octave	9	0.098	0.069	0.086
	6	0.012	0.041	0.007		10	0.068	0.049	0.061
HalfOctave	5	0.052	0.038	0.047		11	0.051	0.036	0.045
	6	0.023	0.016	0.020		12	0.034	0.025	0.031
	7	0.009	0.007	0.008		13	0.025	0.018	0.023
	8	0.006	0.007	0.008		14	0.017	0.013	0.017
	9	0.010	0.015	0.018		15	0.013	0.009	0.012
	10	0.019	0.015	0.017		16	0.007	0.005	0.006
	11	0.035	0.031	0.016		17	0.009	0.006	0.008
Smooth-HalfOctave	6	0.051	0.036	0.045		18	0.007	0.007	0.008
	7	0.022	0.016	0.020		19	0.012	0.013	0.015
	8	0.010	0.008	0.009		20	0.019	0.021	0.026
	9	0.008	0.005	0.006		21	0.027	0.026	0.032
	10	0.006	0.010	0.012		22	0.023	0.022	0.027
	11	0.014	0.019	0.023		23	0.018	0.038	0.036
	12	0.016	0.018	0.022		24	0.043	0.064	0.069
	13	0.027	0.021	0.007		25	0.023	0.027	0.001

## 5 Discussion

### 5.1 The characteristics of the four types of CSP

The simulation and experimental results of this paper clearly show the characteristics of PBOF using the four types of CSP, which are as follows:

- Whether when measuring the micro-displacement (0~1 pixels) and large-scale displacement (5~9 pixels) or in the simulated entire displacement interval (0~9 pixels), the accuracy that can be achieved by using the earliest proposed Octave CSP is at the lowest level among the four types of CSP.
- The comprehensive performance of PBOF using the HalfOctave CSP is the best. The HalfOctave CSP is the highest in the micro-displacement measurement accuracy and the average accuracy of the entire displacement interval.
- When measuring micro-displacement, the accuracy obtained using the SmoothHalfOctave CSP is relatively low. The accuracy achieved using the SmoothHalfOctave CSP is medium when measuring large-scale displacement. The overall measurement accuracy obtained using the SmoothHalfOctave CSP is low in the simulated entire displacement interval.
- When measuring micro-displacement or in the simulated full displacement interval, the accuracy obtained using the QuarterOctave CSP is at a medium level. However, a higher accuracy can be obtained when measuring large-scale displacement using the QuarterOctave CSP.

### 5.2 Problems that need attention in practical applications

No matter which pyramid is used, PBOF using the optimal level filter is high in accuracy and has the most considerable effective displacement measurement interval. Therefore, finding and using the optimal level filter when PBOF using the four types of CSP is applied in engineering practice is essential. In addition, the law of translation motion displacement measurement in Section 4.3 is almost the same as that of vibration parameter measurement in Section 4.4. Therefore, for the vibration parameter measurement occasions that do not require a direct measure of displacement, the selection of CSP types and filter levels should be based on their capability of accurately measuring the displacement caused by vibration.

Although PBOF using all CSPs can achieve effective measurement (with RE being less than 10%) in the displacement interval of the simulation, the RE corresponding to the four pyramids is less than 2% with higher accuracy when the displacement is less than 5 pixel (Fig 13). Therefore, the displacement measurement interval can be controlled below 5 pixels as far as possible through system design in practical applications.

According to the characteristics of PBOF using different CSPs, the HalfOctave pyramid can be given priority in the cases of considerable displacement variation and micro-displacement measurement. PBOF using this CSP has the highest overall accuracy in the whole effective displacement measurement interval simulated in this study, and the accuracy is also the highest when measuring the micro-displacement of less than 1 pixel. In large-scale displacement measurement occasions, the QuarterOctave CSP can be given priority, and PBOF using this CSP has the highest accuracy when measuring large-scale displacement.

## 6 Conclusions

The CSP performs well when applied to phase-based Euler motion amplification, but the impact of its filter types and parameters on motion parameter measurement performance hasn’t been systematically studied. In this paper, the performance of the PBOF using Octave, HalfOctave, SmoothHalfOctave, and QuarterOctave CSP to measure motion parameters is systematically evaluated from three aspects: effective displacement measurement interval, accuracy within effective displacement measurement interval, and vibration parameter measurement accuracy. The main conclusions are as follows:

- The optimal level filter of each of the four types of CSP is at about 1/2 of the effective level range. When using the optimal level filter, PBOF has the highest accuracy and the most considerable effective displacement measurement interval.
- Among the four types of CSP, PBOF using the HalfOctave CSP has the best comprehensive performance and can be the first choice for most applications.
- As for the vibration parameter measurement occasions that do not require directly measuring the displacement, the selection of CSP types and filter levels should also be based on their capability to measure the displacement generated by the vibration accurately.

PBOF using the CSP is a relatively new visual displacement measurement method. At present, the optimization of this method lags behind that of other mainstream visual displacement measurement methods, and there is still much room for improvement in terms of overall framework design, sub-pixel estimation, etc. Meanwhile, as mentioned in the literature [31], this type of phase-based motion estimation using the CSP could be easily integrated with signal processing methods, such as blind source separation, to realize the analysis of structural dynamic characteristics under field conditions. The displacement measurement accuracy of PBOF is not only affected by the types and parameters of CSP filters, but also by factors such as visual marker type, image noise, and image contrast. In-depth study of the impact of these factors upon the displacement measurement accuracy of PBOF will be also the direction of the next step.
